# A Breast Cancer Survivor’s Self-Controlled Case Report: Methylprednisolone Acetate Provided a Week Longer Analgesia Than Dexamethasone Sodium Phosphate via Thoracic Paravertebral Blockade

**DOI:** 10.7759/cureus.6085

**Published:** 2019-11-06

**Authors:** Jinlei Li, Kay Lee, Daniel Chang, Praba Boominathan, Trevor Banack

**Affiliations:** 1 Anesthesiology, Yale School of Medicine, New Haven, USA; 2 Anesthesiology, Montefiore Medical Center, New York, USA

**Keywords:** paravertebral block, glucocorticoid, methylprednisolone acetate, adjuvant, breast cancer, lipophilic glucocorticoids

## Abstract

Proper perioperative pain control with opioid-sparing techniques that extend into post-discharge arena is desirable yet hard to accomplish in breast cancer patients. We here reported a case where we took advantage of long-acting local anesthetics in conjunction with glucocorticoids of different hydrophilic/lipophilic properties and achieved prolonged analgesia for days after single administration thoracic paravertebral blockade. Further exploration into the potential effects of long-acting glucocorticoids in breast cancer patients through peripheral nerve blockage is warranted.

## Introduction

The potential negative roles of opioids on breast cancer pain control and survival have been implicated at multiple levels including involvement in less apoptosis, enhanced tumor angiogenesis/progression, and increased tumor cell resistance to chemotherapy [1-5]. Multiple techniques have been employed to minimize opioid usage perioperatively [6], but once discharged, many patients are left with opioids as the mainstay for pain management. As a result, even though much attention has been directed toward opioid epidemic and minimization of opioids while in hospital, not much is planned or implemented once patient is home. The transition from acute pain to chronic pain is complexed and poorly understood, but surgery-induced pain sensitization appears to be an integral part. While it is still controversial regarding the roles of regional anesthesia in the development of persistent postsurgical pain, it is hard to image for a patient to have persistent surgical pain or chronic opioid usage after a surgery if a patient has prolonged opioid-free analgesia and used very little if any opioids perioperatively. We here reported a unique way to offer the patient with longer than a week of opioid-sparing analgesia by utilizing chronic pain management techniques in acute pain setting. This work has been reported in line with the SCARE criteria [7].

## Case presentation

A 69-year-old female, with height 5’6” and weight 56.7 kg, without significant past medical history, originally presented for bilateral skin-sparing mastectomies/right-sided axillary lymph node dissection/expander placement due to right-sided breast lobular carcinoma.

Bilateral T4 single-injection paravertebral blocks were performed preoperatively using 3.5 cm 22G TuohyTM needles under standard landmark technique with 20 ml 0.2% ropivacaine and 20 mg methylprednisolone acetate on each side. General anesthesia with an endotracheal tube was induced. During this 7.5-hour-surgery, the patient received 300 mcg fentanyl, 1.4 mg hydromorphone, 50 mg ketamine, and 165 mcg dexmedetomidine. Upon emergence, the patient reported no pain. She continued to have no pain until postoperative day (POD) 2 on discharge. She was discharged home with a 10-day course of pregabalin 100 mg twice daily, diazepam 5 mg every eight hours as needed, and oxycodone 5 mg (total 30) every four hours as needed. Telephone correspondence with the patient revealed that she continued to have no need for oxycodone at home until POD 7 when diffuse pain slowly came back. She experienced such a high level of satisfaction with the paravertebral block that before her subsequent bilateral implant exchange (four months after the original surgery), she called the anesthesia department to ensure she would receive the same block.

For the second surgery, she indeed received bilateral T4 paravertebral blocks under the same technique except with 20cc 0.25% bupivacaine and 4 mg dexamethasone sodium phosphate on each side. General anesthesia with endotracheal tube was again induced and the patient received 150 mcg fentanyl and 0.4 mg hydromorphone intraoperatively for the 1.5-hour surgery. She was discharged home on the day of surgery with tramadol 50 mg (total 30) every six hours as needed. On telephone follow-up, the patient stated that she needed to take opioids much earlier, on POD 1 following the secondary surgery instead of POD 7 in the original surgery, to be precise. She had no residual sensory or motor deficits after block wore off for each surgery.

## Discussion

Chronic pain is one of the major devastating complications after surgery, and common procedures such as mastectomy have an incidence of persistent postsurgical pain in up to 20%-50% of the patients. The development of persistent postsurgical pain is multifactorial, including but not limited to preoperative opioid usage, underlying psychosocial factors, intraoperative nerve injury, severe perioperative pain that is poorly controlled, and persistent inflammation. Regional anesthesia and glucocorticoids are both implicated in pharmacological interventions on the transition from acute to chronic pain. Regional anesthesia in the format of central and peripheral nerve blockade can offer opioid-sparing analgesia, but the pain control duration is limited to less than 24 hours unless an indwelling catheter is in place.

Several classes of local anesthetic adjuvants have been used to prolong and augment regional anesthesia. In particular, glucocorticoids have proven to be a useful adjunct to local anesthetics in central and peripheral nerve blocks [8,9], with potential mechanisms including anti-inflammatory properties, immune-modulating effects, classical genome-dependent and newly identified non-genome-dependent glucocorticoid receptor expression, and subsequent modulation on nociception [8]. Non-particulate glucocorticoid dexamethasone sodium phosphate is the most commonly employed glucocorticoid type of local anesthetic adjuvant that prolongs nerve blockade by approximately six to eight hours [10]. Methylprednisolone acetate has been safely and successfully used for neuropathic pain management and chronic pain treatment with effects last from days to weeks, via intrathecal, epidural paravertebral route of injection, peripheral nerve blockade, or joint injection [11-17]. There are no studies in the current literature comparing the effects of hydrophilic/non-particulate versus lipophilic/particulate glucocorticoids in peripheral nerve blocks.

To our best knowledge, the case described above is the first one in this regard and it is unique in that a breast cancer survivor served as her own control. The prolongation of blockade by methylprednisolone acetate was likely due to the lipophilic feature, exhibiting a depot-type slow release. Comparing the lipophilic glucocorticoids, such as methylprednisolone acetate, versus hydrophilic ones, such as dexamethasone sodium phosphate, in acute pain control within perioperative arena would be an interesting area of further research, particularly from the perspectives of analgesia duration, opioid consumption, length of hospital stays, patient satisfaction, and potential roles in the prevention of transition from acute pain to chronic pain.

## Conclusions

The management of acute pain after a surgical procedure does not end when a patient is discharged from the hospital, rather it is the responsibility of perioperative physicians, surgeons, and anesthesiologists alike, until the resolution of the acute pain, with lasting effects on persistent postsurgical pain prevention. There are readily chronic pain management techniques such as using long-acting glucocorticoids with demonstrated safety track record, and it is easy to implement, as shown in this case report. The thoughtful combination of acute and chronic pain techniques in perioperative arena could be a new direction for comprehensive acute surgical pain management.
